# 6-month symptom changes and factors associated with treatment response following combined acupuncture, moxibustion, and cupping protocol in patients with primary tinnitus: a retrospective cohort study

**DOI:** 10.3389/fneur.2026.1869226

**Published:** 2026-07-14

**Authors:** Yi Zhu, Ke Fang, Lianqiang Fang, Jie Zhou, Minghui Zhao, Hantong Hu, Da Jiang, Hong Gao, Yang Li

**Affiliations:** 1 The Third Clinical College of Zhejiang Chinese Medical University, Hangzhou, China; 2 Department of Acupuncture and Moxibustion, The Third Affiliated Hospital of Zhejiang Chinese Medical University, Hangzhou, China

**Keywords:** acupuncture, combined TCM intervention, factors associated with meeting response criteria, long-term changes in THI scores, retrospective cohort study, tinnitus

## Abstract

**Background:**

Primary tinnitus is a highly prevalent auditory disorder, affecting approximately 14.4% of adults globally. Conventional interventions yield limited efficacy. Although acupuncture-based TCM interventions is widely applied for tinnitus management, robust evidence regarding 6-month sustained symptom changes following combined TCM intervention and factors associated with treatment response remains scarce.

**Objective:**

To explore short- and long-term (6-month) symptom changes following standardized combined acupuncture, moxibustion, and cupping therapy in patients with primary tinnitus, and to analyze factors associated with treatment response.

**Methods:**

We conducted a single-center retrospective cohort study at the Third Affiliated Hospital of Zhejiang Chinese Medical University, enrolling 140 patients with primary tinnitus who received standardized combined acupuncture, moxibustion, and cupping therapy between January 2024 and August 2025 and completed 6-month follow-up. The primary outcome was a ≥30% reduction in Tinnitus Handicap Inventory (THI) score from baseline; the secondary outcome was a ≥1-grade reduction in THI severity grade. Univariate screening followed by binary logistic regression was used to identify factors associated with treatment response.

**Results:**

All 140 patients completed follow-up. The primary response rate was 57.1% (80/140) at treatment completion and increased to 81.4% (114/140) at the 6-month follow-up. The secondary response rate rose from 63.6% post-treatment to 82.9% at follow-up. Multivariate regression showed that younger age (OR = 0.577, *P* = 0.028) and higher baseline THI grade (OR = 1.662, *P* = 0.008) were associated with meeting response criteria in the short term. However, neither association remained significant in sensitivity analyses excluding acute tinnitus cases (age: OR = 0.683, 95% CI 0.378–1.236, *P* = 0.208; THI grade: OR = 1.486, 95% CI 0.927–2.380, *P* = 0.100). For long-term observed THI score changes, both age (OR = 0.326, *P* = 0.003) and baseline THI grade (OR = 2.699, *p* = 0.001) were associated with meeting the primary response criterion; baseline THI grade (OR = 4.455, *p* < 0.001) and age (OR = 0.266, *P* = 0.001) predicted the secondary outcome. Gender, tinnitus laterality, disease duration, and comorbid symptoms showed no significant effects.

**Conclusion:**

Reductions in tinnitus-related handicap were observed over 6 months following combined acupuncture, moxibustion, and cupping therapy. Factors associated with treatment response varied by timepoint: for long-term changes, baseline THI severity and age were associated with greater observed score reductions; short-term associations for these factors were not robust in sensitivity analyses.

## 1 Introduction

Primary tinnitus refers to an abnormal auditory sensation perceived subjectively by an individual in the absence of external sound stimulation, often presenting as hissing, sizzling, or ringing sounds that may be persistent or intermittent. Some patients may experience multiple combined sounds (1). Clinically, tinnitus is categorized by duration: tinnitus lasting ≤ 3 months is defined as acute tinnitus, while tinnitus persisting more than 3 months is defined as chronic tinnitus (2). The global prevalence of tinnitus in adults is approximately 14.4% (about 740 million people), among whom 2.3% (over 120 million people) suffer from significant functional impairment due to severe tinnitus (3). Its pathogenesis has not been fully elucidated and may be related to otological factors such as hearing loss, occupational noise exposure, use of ototoxic drugs (e.g., platinum-based agents), and otitis media, as well as non-otological factors including temporomandibular joint disorders and depression (4, 5).

Numerous treatment modalities are available for primary tinnitus, including cognitive behavioral therapy (CBT), pharmacological agents (e.g., lidocaine, anticonvulsants, Ginkgo biloba, and beta-blockers), sound therapy (hearing aids, tinnitus maskers, auditory stimulation, and auditory training) (6), and non-invasive brain stimulation (7). Despite these interventions, current management remains suboptimal, and safer and more beneficial intervention strategies are still needed. Within the clinical practice of China, acupuncture has been widely recognized as a safe and beneficial intervention for tinnitus (8).

Acupuncture has a long history in the treatment of tinnitus in China, with the earliest medical records dating back to the 5th century BCE (9). Both clinical studies and animal experiments have confirmed that acupuncture exerts beneficial effects on primary tinnitus (10–12), and acupuncture combined with Western medicine is superior to Western medicine alone in improving tinnitus severity and remission rate (13). However, the response to acupuncture is influenced by multiple factors such as THI severity, age, gender, and disease duration, which have been insufficiently explored in existing studies. In addition, most studies focus on short-term changes in THI scores and lack systematic evaluation of long-term changes in THI scores (e.g., over 6 months).

Therefore, this single-center retrospective cohort study enrolled 140 patients with primary tinnitus who received standardized combined acupuncture, moxibustion, and cupping therapy and completed 6-month follow-up. Using the Tinnitus Handicap Inventory (THI) as the core assessment tool, we systematically evaluated the short-term (post-treatment) and long-term (6-month follow-up) symptom changes of the combined TCM intervention for tinnitus. We also explored the impacts of THI severity, age, gender, disease duration and other factors on meeting response criteria, and screened factors associated with treatment response, so as to provide evidence-based support for the formulation of individualized combined TCM intervention protocols for primary tinnitus.

## 2 Materials and methods

### 2.1 Subjects

A single-center retrospective cohort study was conducted at the Department of Acupuncture, Third Affiliated Hospital of Zhejiang Chinese Medical University. The treatment period was January 2024 to August 2025, with a 6-month follow-up after treatment completion. Data quality was ensured via electronic medical record review, dual independent data entry, and follow-up verification. The study was approved by the hospital ethics committee (Approval No.: ZSLL-ZN-2025-071).

### 2.2 Inclusion and exclusion criteria

#### 2.2.1 Diagnostic criteria

Diagnosis of primary tinnitus was based on *Practical Otorhinolaryngology Head and Neck Surgery* (14), with criteria consistent with the 2014 American Academy of Otolaryngology–Head and Neck Surgery Foundation (AAO-HNSF) Clinical Practice Guideline (4) and the 2022 German S3 Guideline on Chronic Tinnitus (2), patients were diagnosed when they met all of the following:(1) Tinnitus as the chief complaint, absent external sound source; (2)Tinnitus disturbs daily life, work, or sleep; (3) Accompanied by insomnia, irritability, anxiety, or depression; (4) With or without sensorineural hearing loss; (5) Organic ear or neurological lesions excluded by otoscopy, audiometry, with imaging.

As part of the comprehensive otologic evaluation to exclude secondary causes of tinnitus, all patients underwent pure-tone audiometry (PTA) by certified audiologists. Hearing loss, when present, was defined as a four-frequency pure-tone average (4fPTA) >20 dB HL across 0.5, 1, 2, and 4 kHz in the better-hearing ear, consistent with the WHO hearing impairment grading system (15), notably, hearing status was not an inclusion criterion; patients were eligible regardless of the presence or absence of hearing loss, provided that primary tinnitus was confirmed and structural or neurological causes were excluded.

#### 2.2.2 Inclusion criteria

The inclusion criteria were as follows: (1) Met the above diagnostic criteria; (2) Age > 10 years, any gender; (3) Tinnitus duration was recorded but not restricted; patients with both acute (≤3 months) and chronic (>3 months) primary tinnitus were eligible; (4) Completed one full course (10 sessions) of combined TCM intervention; (5) Able to understand and comply with assessments and follow-up; (6) Baseline THI grade ≥Grade 2.

#### 2.2.3 Exclusion criteria

The exclusion criteria were as follows: (1) Pregnancy or lactation; (2) Tinnitus secondary to structural ear disease (e.g., canal atresia, ossicular discontinuity, vestibular schwannoma); (3) Severe systemic disease or organ failure; (4) Major psychiatric disorder precluding cooperation; (5) any condition requiring further imaging (temporal bone CT and/or brain MRI) for clinically indicated reasons, including asymmetric sensorineural hearing loss, pulsatile tinnitus, focal neurological signs, history of head trauma, or suspected other retrocochlear lesions.

#### 2.2.4 Criteria for shedding, removal and suspension

The attrition criteria were as follows: patients were excluded if: misclassified, lost to follow-up, non-adherent, or developed serious adverse events.

### 2.3 Treatment methods

**Figure 1:**
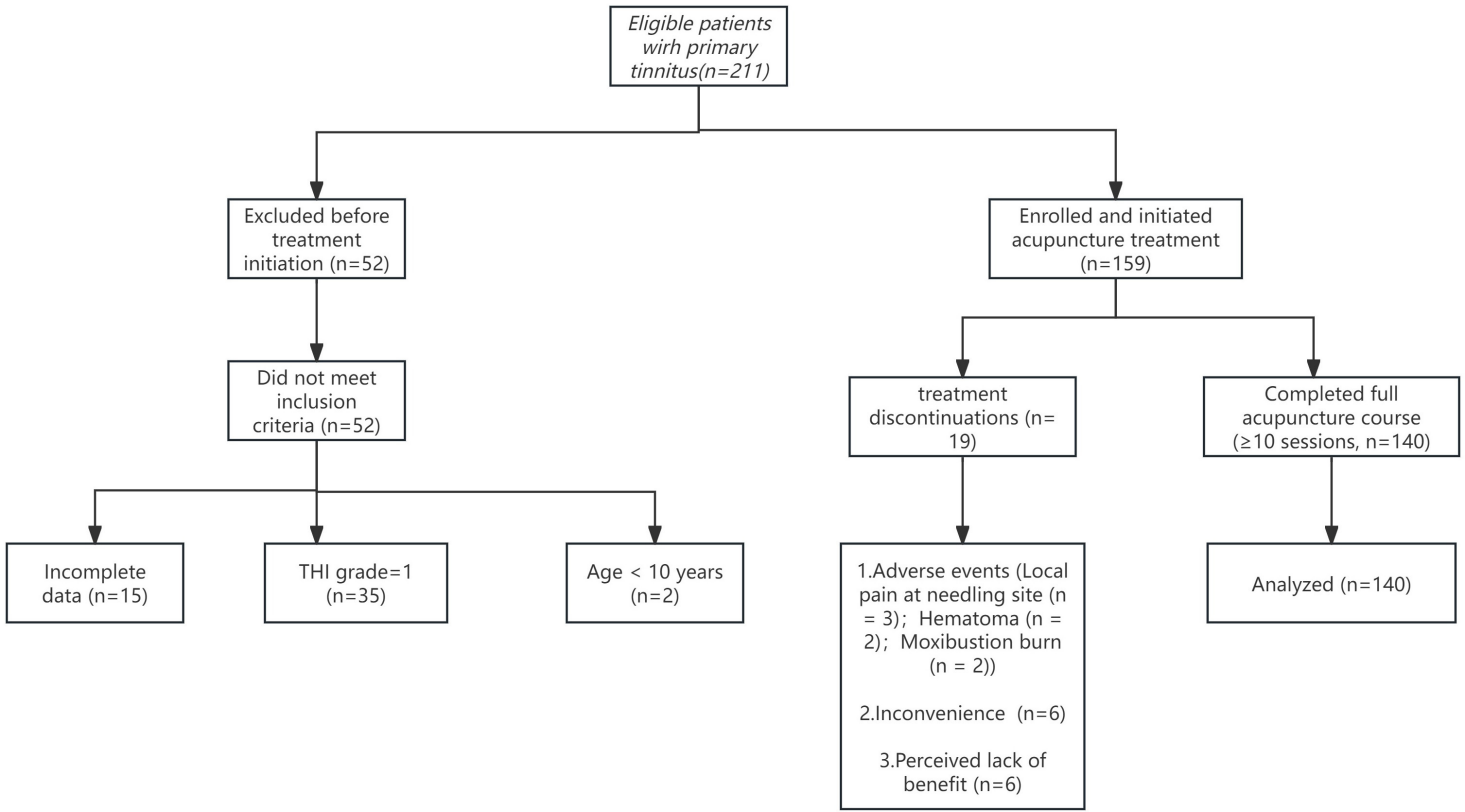
Flowchart of participant screening and enrollment.

A total of 211 patients with primary tinnitus were screened during the study period. Before treatment initiation, 52 patients were excluded, including 15 cases with incomplete clinical data, 35 cases with THI Grade I, and 2 cases aged under 10 years. The remaining 159 patients initiated the full course of standardized combined TCM intervention. Among them, 19 patients were discontinued treatment before completion, with treatment discontinuation related to adverse events (three suffered transient local acupuncture-site pain, two developed small hematomas at Yifeng, and two sustained mild erythematous earlobe burns from moxibustion; all symptoms resolved rapidly with simple management), Inconvenience, Perceived lack of benefit. Ultimately, 140 patients who completed 10 combined TCM intervention sessions and the 6-month follow-up assessment were included in the final analysis (Figure 1).

The intervention evaluated in this study was a standardized combined Traditional Chinese Medicine (TCM) protocol consisting of three components delivered sequentially in each session: (1) manual acupuncture, (2) moxibustion, and (3) retroauricular flash cupping. All three components were administered to every participant as an integrated intervention; the specific contributions of the individual components (acupuncture, moxibustion, or cupping) to the observed outcomes cannot be isolated or distinguished in this retrospective study design.

Combined TCM intervention rationale:the combined TCM intervention was based on Traditional Chinese Medicine (TCM) style acupuncture, with the treatment rationale grounded in TCM theory and clinical practice guidelines.

Therapy: (1) Manual acupuncture.

**Figure 2:**
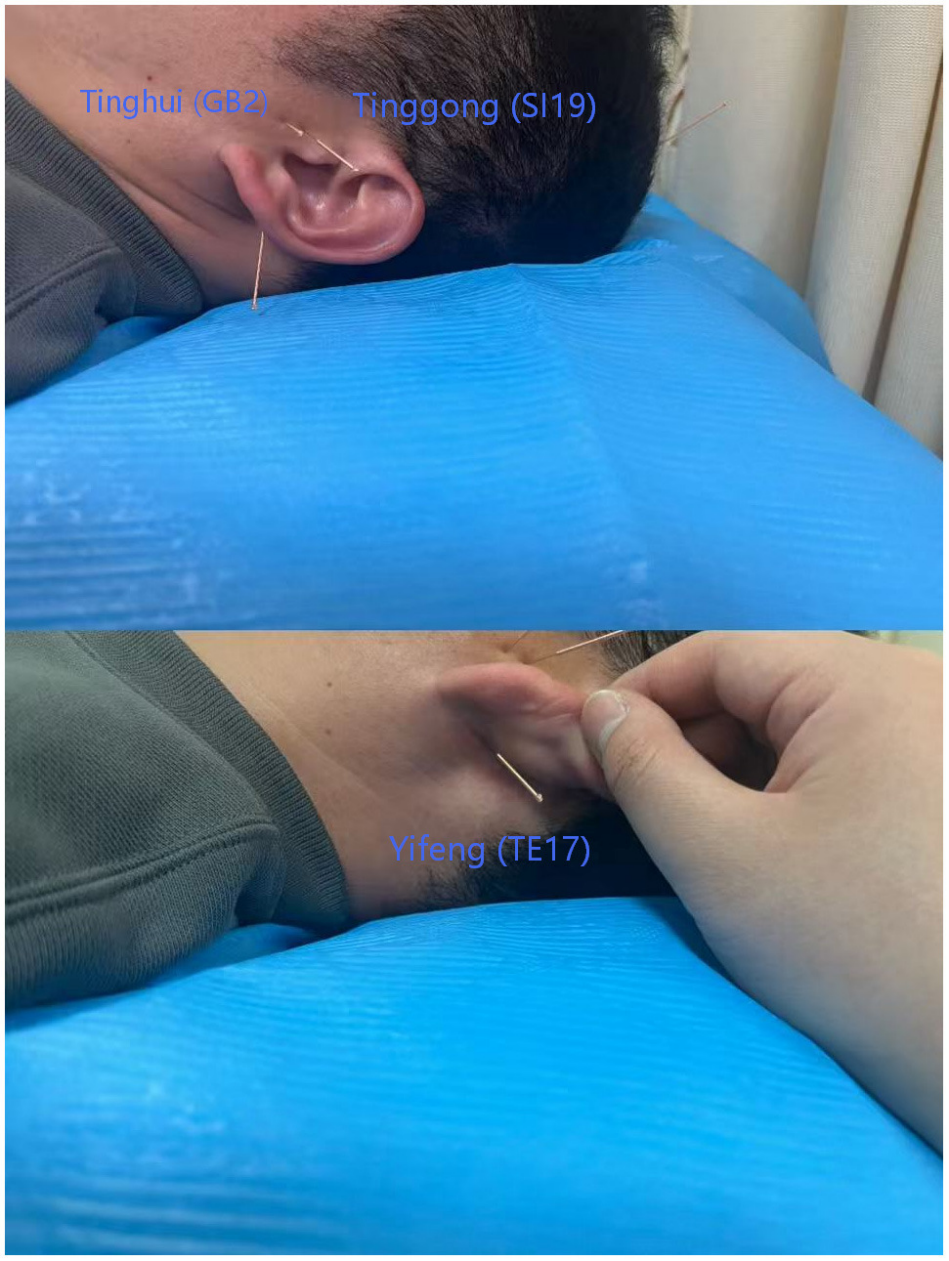
Manipulation of three periauricular acupoints in patients with primary tinnitus.

Acupoint selection: Tinggong (SI19), Tinghui (GB2), and Yifeng (TE17). For unilateral tinnitus, the affected side only; for bilateral tinnitus, both sides (Figure 2).

Operation: Sterile 0.30 mm × 50 mm needles were inserted perpendicularly to a depth of 20–30 mm to elicit *de qi* (soreness, fullness, or radiation toward the ear). During the 30-min needle retention period (16) (Table 1), the Qi-guiding and collateral-unblocking manipulation was applied at the three periauricular acupoints to maintain needling sensation and directional conduction of qi. Moxibustion was performed concurrently while the needles were retained *in situ*.

**Table 1:** Location of three periauricular acupoints in patients with primary tinnitus.

Acupoint name	Location criteria
**Tinggong (SI19)**	In the depression between the midpoint of the tragus and the mandibular condyle
**Tinghui (GB2)**	In the depression between the intertragic notch and the mandibular condyle
**Yifeng (TE17)**	Behind the earlobe, in the depression anterior to the lower tip of the mastoid process

(2) Moxibustion.

Following elicitation of *de qi* at the three periauricular acupoints, a section of moxa stick (diameter approximately 1.8 cm, length approximately 2 cm) was ignited from the lower end and allowed to burn out naturally (one *zhuang* in total).

(3) Retroauricular flash cupping.

After needle removal and completion of moxibustion, patients remained in a sitting position with retroauricular skin fully exposed. Flash cupping was applied locally around the mastoid process using a No. 2 glass cup (mouth diameter approximately 3.0 cm) until the local skin became flushed.

Protocol: The protocol was standardized, with no variation in acupoint selection or stimulation method across participants. Treatment was administered every other day, 3 sessions per week, with 10 sessions constituting one course. The full course of 10 treatment sessions took about 4 weeks to complete. All therapies were performed in the hospital outpatient department following a unified standard operating procedure, and routine precautions were told to all patients.

Practitioner details: The combined TCM intervention was delivered by a single practitioner, who is a senior chief physician with 30years of clinical experience in combined TCM intervention for tinnitus and holds licensure in TCM acupuncture from the Zhejiang Provincial Health Commission. Prior to the study, the practitioner was trained in the standardized protocol to ensure consistency. Patients with unilateral tinnitus received needles only on the affected side, while bilateral tinnitus patients received bilateral needling, which led to different needle counts and treatment doses. This factor (tinnitus laterality / varying treatment dose) was included in the univariate and multivariate analyses in the present study. It should be noted that the specific contributions of moxibustion and cupping to these observed THI score changes cannot be isolated in this retrospective study.

### 2.4 Follow-up

6 months after treatment completion, two trained investigators administered the THI via telephone. The follow-up assessment content was identical to that recorded at the baseline visit. It is worth noting that this is a real-world retrospective study. Routine clinical medical records did not systematically document whether patients received additional tinnitus-related interventions, including pharmacological treatment, hearing aids, sound therapy, tinnitus retraining therapy (TRT) and cognitive behavioral therapy (CBT) during combined TCM intervention treatment and the 6-month follow-up period. Therefore, the influence of these potential concurrent interventions cannot be fully excluded.

### 2.5 Outcome measures

Response to combined TCM intervention was comprehensively evaluated using the Tinnitus Handicap Inventory (THI). Assessments were performed at three time points: baseline, immediately post-treatment, and at 6-month follow-up. The culturally adapted Chinese version of the Tinnitus Handicap Inventory (THI) was used to measure tinnitus-related handicap. This scale was translated and culturally adjusted following international standards. Previous studies have proven that the Chinese THI has good reliability (Cronbach's α = 0.93) and validity in Chinese tinnitus patients, and is suitable for clinical outcome assessment (17). The THI is a 25-item self-reported questionnaire with a total score ranging from 0 to 100 points, where higher scores indicate greater tinnitus-related handicap and functional impairment. Based on the total score, tinnitus severity is stratified into five grades: Grade I (0–16 points, no handicap), Grade II (18–36 points, mild handicap), Grade III (38–56 points, moderate handicap), Grade IV (58–76 points, severe handicap), and Grade V (78–100 points, catastrophic handicap) (18).

The primary outcome was defined as a ≥30% reduction in THI score from baseline at the 6-month follow-up. The ≥30% reduction threshold for defining meeting response criteria is grounded in established psychometric and clinical reasoning. The minimal clinically important difference (MCID) for the Tinnitus Handicap Inventory (THI) has been empirically determined at 7 points (approximately 7% of the 0–100 scale range) based on anchor-based analyses correlating score changes with patient-reported global impression of improvement, corroborated by distribution-based methods (half standard deviation, effect size d = 0.5) (19) For subjective chronic conditions such as tinnitus and chronic pain, improvements of ≥15% of the total scale range are considered clinically meaningful as a conservative rule of thumb (20). Our 30% criterion therefore represents a doubling of this conservative benchmark, ensuring that classified responders experienced not merely minimal perceptible improvement but a substantial, clinically robust reduction in tinnitus-related handicap. However, we acknowledge that this relative percentage-change outcome is inherently baseline-dependent and may introduce mathematical coupling when baseline THI grade is simultaneously included as a predictor variable. To address this limitation, we conducted supplementary analyses using an absolute THI change criterion (≥7 points) as a sensitivity check (Supplementary Table 10).

The secondary outcome was assessed based on changes in THI grade from baseline to 6-month follow-up, categorized into four levels: complete symptom relief: Complete resolution of tinnitus and associated symptoms, with no relapse for ≥1 month; Markedly effective: Reduction in THI grade by ≥2 grades; Effective: Reduction in THI grade by 1 grade; Ineffective: No change in THI grade (21, 22). And the overall response proportion was calculated as follows:


$$\begin{array}{l} \text{Total effective rate }(\%)\\ \;=\frac{\text{Number of cured }+\text{ markedly effective }+\text{ effective cases}}{\text{Total number of cases}}\\ \;\times 100\%. \end{array}$$

### 2.6 Statistical analysis

All statistical analyses in this study are exploratory and hypothesis-generating. Analyzed with SPSS 26.0. Continuous data: mean ± SD, *t*–test. Categorical data: *n* (%), χ^2^ or Fisher exact test. Variables with *P* < 0.05 in univariate analysis were entered into binary logistic regression to identify factors associated with treatment response. Age groups were coded as ordinal categories: ≤ 39 years = 1, 40–59 years = 2, and ≥60 years = 3. THI severity grades were coded as 1 (Grade II), 2 (Grade III), 3 (Grade IV), and 4 (Grade V). Both variables were treated as ordinal predictors in the logistic regression models. *P* < 0.05 was considered statistically significant. Model fit was assessed with the Hosmer–Lemeshow test. Covariates for binary logistic regression were selected based on both clinical relevance and univariate analysis, instead of statistical significance alone, to prevent exclusion of clinically important factors.

#### 2.6.1 Descriptive statistics

Categorical variables (including gender, tinnitus laterality, comorbid symptoms, age, THI score, and disease duration) are presented as counts (proportions) *n* (%).

#### 2.6.2 Response analysis

Based on the THI scores at the end of treatment and at the 6-month follow-up, the reduction rate in THI scores was calculated. A reduction of ≥30% in the THI score was defined as meeting the response criterion; otherwise, it was defined as not meeting the criterion. Concurrently, hierarchical response analysis was conducted based on changes in tinnitus severity grades, and the overall response proportion was calculated.

#### 2.6.3 Influencing factor analysis via binary logistic regression

Efficacy at the end of treatment (effective = 1, ineffective = 0) and at the 6-month follow-up (effective = 1, ineffective = 0) were used as dichotomous dependent variables, respectively. Independent variables were first screened using univariate chi-square tests. Variables with *P* < 0.05 in the univariate analysis were included in the model to construct binary logistic regression models. Regression coefficients (β), standard errors (SE), *P*-values, and odds ratios (OR) were reported to identify factors associated with meeting response criteria for the short- and long-term changes in THI scores of combined TCM intervention in the treatment of primary tinnitus.

#### 2.6.4 Model diagnostics

The Hosmer-Lemeshow (HL) test was used to assess the goodness-of-fit of the logistic regression models. For the binary logistic regression analysis using the primary outcome at the end of treatment as the dependent variable: χ^2^ = 7.447, df = 8, *P* = 0.489 > 0.05; For the analysis using the secondary outcome at the end of treatment: χ^2^ = 8.694, df = 8, *P* = 0.369 > 0.05; For the analysis using the primary outcome at follow-up: χ^2^ = 5.119, df = 8, *P* = 0.745 > 0.05; For the analysis using the secondary outcome at follow-up: χ^2^ = 2.895, df = 8, *P* = 0.941 > 0.05. These results indicated good model fit for all analyses.

#### 2.6.5 Sensitivity analysis

Given that 37.1% (52/140) of participants had tinnitus duration ≤ 3 months (acute), which may show spontaneous improvement or regression to the mean, we performed a sensitivity analysis excluding these patients. Binary logistic regression was repeated in the chronic tinnitus subgroup (>3 months, n=88) with identical model specifications. These results show that age and baseline THI grade are not relevant factors for short-term symptom improvement among the included patients. Different from short-term results, the associations of age and THI grade remained statistically significant in the long-term analysis across both the original and sensitivity datasets (Supplement Table 8).

#### 2.6.6 Multicollinearity analysis

Binary logistic regression was used to identify factors associated with treatment response. Multicollinearity among independent variables was assessed using the variance inflation factor (VIF). All VIF values were between 1.20 and 1.45, well below the conventional threshold of 5, indicating no significant multicollinearity in the multivariable models.

#### 2.6.7 Formal assessment of the linearity assumption

The linearity assumption for ordinal predictors (age group and baseline THI grade) was formally assessed using likelihood ratio tests comparing ordinal coding models vs. dummy variable models for all four logistic regression models. For the short-term primary outcome (≥30% THI reduction), the LRT was non-significant (ordinal coding:−2LL = 184.597, 2 parameters; dummy variable coding:−2LL = 181.869, 5 parameters; χ^2^ = 2.728, df = 3, *P* = 0.435). For the short-term secondary outcome (≥1-grade THI reduction), the LRT was also non-significant (ordinal coding:−2LL = 169.998, 2 parameters; dummy variable coding:−2LL = 168.144, 5 parameters; χ^2^ = 1.854, df = 3, *P* = 0.604). For the long-term primary outcome (≥30% THI reduction), the LRT was non-significant (ordinal coding:−2LL = 113.256, 2 parameters; dummy variable coding:−2LL = 110.665, 5 parameters; χ^2^ = 2.591, df = 3, *P* = 0.459). For the long-term secondary outcome (≥1-grade THI reduction), the LRT was also non-significant (ordinal coding:−2LL = 99.260, 2 parameters; dummy variable coding:−2LL = 96.638, 5 parameters; χ^2^ = 2.622, df = 3, *P* = 0.454). This supports the appropriateness of the ordinal coding assumption.

## 3 Results

### 3.1 Baseline characteristics

A total of 140 patients were included: 42 males (30.0%) and 98 females (70.0%). Age distribution: ≤ 39 years (50, 35.7%), 40–59 years (66, 47.1%), and ≥60 years (24, 17.1%). THI grade distribution: Grade II (43, 30.7%), Grade III (49, 35.0%), Grade IV (29, 20.7%), and Grade V (19, 13.6%). Disease duration: ≤ 3 months (52, 37.1%), >3 months (88, 62.9%). Laterality: left (38, 27.1%), right (37, 26.4%), bilateral (65, 46.4%). Comorbidities: negative emotions (88, 62.9%), sleep disturbance (90, 64.3%), hearing loss (84, 60.0%), vertigo (33, 23.6%), ear fullness/aural pressure (85, 60.7%), hypertension (35, 25.0%), diabetes mellitus (15, 10.7%), rhinitis (52, 37.1%) (Table 2).

**Table 2:** Baseline clinical characteristics of patients.

Variable	*n* (%)
Gender	
Male	42 (30.0)
Female	98 (70.0)
Age, years	
≤39	50 (35.7)
40–59	66 (47.1)
≥60	24 (17.1)
THI grade	
Grade II	43 (30.7)
Grade III	49 (35.0)
Grade IV	29 (20.7)
Grade V	19 (13.6)
Tinnitus laterality	
Left ear	38 (27.1)
Right ear	37 (26.4)
Bilateral	65 (46.4)
Disease duration	
≤3 months	52 (37.1)
>3 months	88 (62.9)
Associated symptoms	
Negative emotions	88 (62.9)
Sleep disturbance	90 (64.3)
Hearing loss	84 (60.0)
Vertigo	33 (23.6)
Ear fullness	85 (60.7)
Comorbidities	
Hypertension	35 (25.0)
Diabetes mellitus	15 (10.7)
Rhinitis	52 (37.1)

### 3.2 Changes in THI scores at different time points

**Figure 3:**
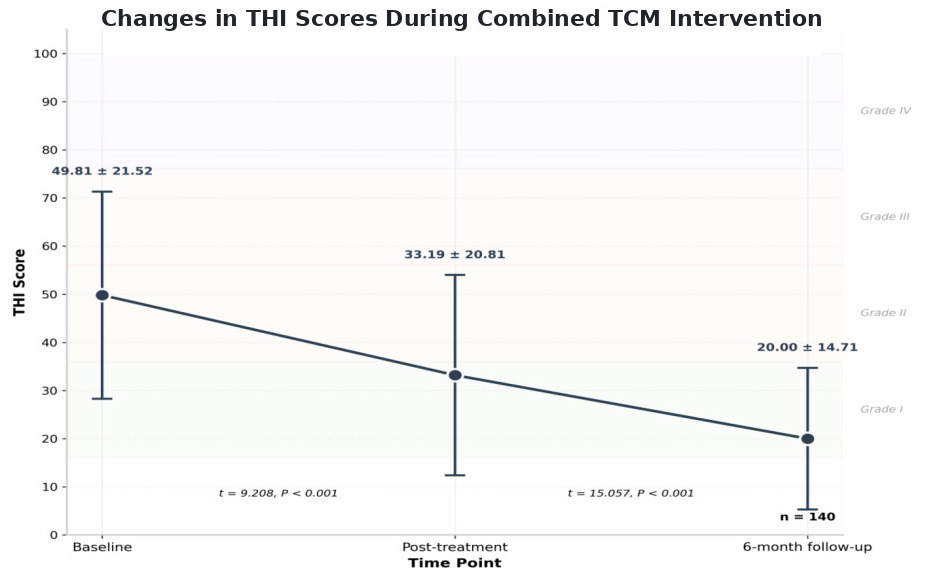
Trend of mean THI scores.

In this part, we independently compared raw THI scores at different time points, which was analyzed separately from the percentage-based efficacy evaluation. The baseline total THI score was 49.81 ± 21.52. Post-treatment, the THI score was 33.19 ± 20.81, representing a mean decrease of 16.62 ± 21.36 from baseline [95% confidence interval (CI): 13.052, 20.191]; this difference was statistically significant (*t* = 9.208, *P* < 0.001). At the 6-month follow-up, the total THI score was 20.00±14.71, with a mean decrease of 29.81 ± 23.42 from baseline (95% CI: 25.893, 33.721), and the difference was statistically significant (*t* = 15.057, *P* < 0.001) (Figure 3).

### 3.3 Short-term changes in THI scores and influencing factors

#### 3.3.1 Short-term response rate

Using a ≥30% reduction in THI score from baseline as the criterion for meeting response criteria, 80 of 140 patients (57.1%) were met this criterion at the end of treatment. Using a ≥1-grade reduction in THI severity as the response criterion: 89 patients showed this response (30 markedly effective, 59 effective) at the end of the combined TCM intervention, with a response proportion of 63.6%.

#### 3.3.2 Binary logistic regression analysis of primary outcome measures

Using a ≥30% reduction in THI score at the end of treatment as the dependent variable, binary logistic regression showed that age was associated with short-term changes in THI scores(OR = 0.577, *P*=0.028), and younger patients were more likely to meet response criteria. However, in the sensitivity analysis excluding patients with acute tinnitus (duration ≤ 3 months, *n* = 88), this association was no longer significant (OR = 0.683, 95% CI 0.378–1.236, *P* = 0.208), indicating that the short-term age finding was not robust (Tables 3, 4; Supplementary Table 8). Supplementary analysis using an absolute THI change criterion (≥7 points) showed that baseline THI grade remained significantly associated with response (OR = 2.055, 95% CI 1.354–3.120, *P* = 0.001), but the association with age was not significant under this criterion (OR = 0.696, 95% CI 0.405–1.194, *P* = 0.188), indicating that the short-term finding was less robust to the choice of outcome definition (Supplementary Table 10).

**Table 3:** Concise summary table about significant predictors of treatment response.

Outcome	Time point	Predictor	β	SE	OR (95% CI)	*P*
Primary (≥30% THI reduction)	Short-term	Age	−0.549	0.250	0.577 (0.353–0.943)	0.028
		Baseline THI grade	—	—	—	—
	Long-term	Age	−1.122	0.379	0.326 (0.155–0.684)	0.003
		Baseline THI grade	0.993	0.310	2.699 (1.469–4.958)	0.001
Secondary (≥1-grade THI reduction)	Short-term	Age	—	—	—	—
		Baseline THI grade	0.508	0.190	1.662 (1.144–2.415)	0.008
	Long-term	Age	−1.325	0.416	0.266 (0.118–0.600)	0.001
		Baseline THI grade	1.494	0.387	4.455 (2.086–9.517)	<0.01

**Table 4:** Results of short-term changes in THI scores analysis for primary outcome measures.

Variable	Effective group (*n* = 80) *n* (%)	Ineffective group (*n* = 60) *n* (%)	Univariate *P*-value	Multivariate analysis				
				β	SE	OR [Exp (B)]	95% CI for OR	*P*-value
Gender			0.709					
Male	23 (28.8%)	19 (31.7%)						
Female	57 (71.2%)	41 (68.3%)						
Age group			0.033	−0.549	0.250	0.577	0.353–0.943	0.028
≤39 years	32 (40.0%)	18 (30.0%)						
40–59 years	40 (50.0%)	26 (43.3%)						
≥60 years	8 (10.0%)	16 (26.7%)						
THI grade			0.711					
Grade II	24 (30.0%)	19 (31.7%)						
Grade III	26 (32.5%)	23 (38.3%)						
Grade IV	17 (21.2%)	12 (20.0%)						
Grade V	13 (16.3%)	6 (10.0%)						
Tinnitus laterality			0.918					
Left ear	22 (27.5%)	16 (26.7%)						
Right ear	22 (27.5%)	15 (25.0%)						
Bilateral	36 (45.0%)	29 (48.3%)						
Disease course			0.419					
≤3 months	32 (40.0%)	20 (33.3%)						
>3 months	48 (60.0%)	40 (66.7%)						
Negative emotions			0.130					
No	34 (42.5%)	18 (30.0%)						
Yes	46 (57.5%)	42 (70.0%)						
Sleep disturbance			0.387					
No	31 (38.8%)	19 (31.7%)						
Yes	49 (61.2%)	41 (68.3%)						
Hearing loss			0.163					
No	28 (35.0%)	28 (46.7%)						
Yes	52 (65.0%)	32 (53.3%)						
Vertigo			0.206					
No	58 (72.5%)	49 (81.7%)						
Yes	22 (27.5%)	11 (18.3%)						
Ear fullness and distension			0.881					
No	31 (38.8%)	24 (40.0%)						
Yes	49 (61.3%)	36 (60.0%)						
Hypertension			0.119					
No	58 (72.5%)	36 (60.0%)						
Yes	22 (27.5%)	24 (40.0%)						
Diabetes mellitus			0.752					
No	72 (90.0%)	53 (88.3%)						
Yes	8 (10.0%)	7 (11.7%)						
Rhinitis			0.920					
No	50 (62.5%)	38 (63.3%)						
Yes	30 (37.5%)	22 (36.7%)						

#### 3.3.3 Binary logistic regression analysis of secondary outcome measures

Using a ≥1-grade reduction in THI grade at the end of treatment as the dependent variable, binary logistic regression showed that baseline THI grade was associated with factor for short-term changes in THI scores (OR = 1.662, *P* = 0.008). For each 1-grade increase in THI grade, the odds ratio of meeting response criteria was 1.662 times that of the original; the more severe the baseline tinnitus, the better the short-term changes in THI scores. However, in the sensitivity analysis excluding patients with acute tinnitus (*n* = 88), this association was no longer significant (OR = 1.486, 95% CI 0.927–2.380, *P* = 0.100), indicating that this short-term finding was also not robust (Tables 3, 5; Supplementary Table 8).

**Table 5:** Results of short-term changes in THI scores analysis for secondary outcome measures.

Variable	Effective group (*n* = 89) *n* (%)	Ineffective group (*n* = 51) *n* (%)	Univariate *P*-value	Multivariate analysis				
				β	SE	OR [Exp (B)]	95% CI for OR	*P*-value
Gender			0.156					
Male	23 (25.8%)	19 (37.3%)						
Female	66 (74.2%)	32 (62.7%)						
Age group			0.121					
≤39 years	35 (39.3%)	15 (29.4%)						
40–59 years	43 (48.3%)	23 (45.1%)						
≥60 years	11 (12.4%)	13 (25.5%)						
THI grade			0.049	0.508	0.190	1.662	1.144–2.415	0.008
Grade II	21 (23.6%)	22 (43.1%)						
Grade III	31 (34.8%)	18 (35.3%)						
Grade IV	22 (24.7%)	7 (13.7%)						
Grade V	15 (16.9%)	4 (7.8%)						
Tinnitus laterality			0.465					
Left ear	25 (28.1%)	13 (25.5%)						
Right ear	26 (29.2%)	11 (21.6%)						
Bilateral	38 (42.7%)	27 (52.9%)						
Disease course			0.152					
≤3 months	37 (41.6%)	15 (29.4%)						
>3 months	52 (58.4%)	36 (70.6%)						
Negative emotions			0.701					
No	32 (36.0%)	20 (39.2%)						
Yes	57 (64.0%)	31 (60.8%)						
Sleep disturbance			0.937					
No	32 (36.0%)	18 (35.3%)						
Yes	57 (64.0%)	33 (64.7%)						
Hearing loss			0.197					
No	32 (36.0%)	24 (47.1%)						
Yes	57 (64.0%)	27 (52.9%)						
Vertigo			0.403					
No	66 (74.2%)	41 (80.4%)						
Yes	23 (25.8%)	10 (19.6%)						
Ear fullness and distension			0.480					
No	33 (37.1%)	22 (43.1%)						
Yes	56 (62.9%)	29 (56.9%)						
Hypertension			0.225					
No	63 (70.8%)	31 (60.8%)						
Yes	26 (29.2%)	20 (39.2%)						
Diabetes mellitus			0.406					
No	78 (87.6%)	47 (92.2%)						
Yes	11 (12.4%)	4 (7.8%)						
Rhinitis			0.732					
No	55 (61.8%)	33 (64.7%)						
Yes	34 (38.2%)	18 (35.3%)						

### 3.4 6-Month long-term changes in THI scores and influencing factors

#### 3.4.1 Long-term response rate

Using a ≥30% reduction in THI score from baseline as the effective criterion: 114 patients met the response criterion at the follow-up endpoint, with a response proportion of 81.4%. Using a ≥1-grade reduction in THI severity as the effective criterion: 116 patients showed this response (6 cured, 64 markedly effective, 46 effective) at the follow-up endpoint, with a response proportion of 82.9%.

#### 3.4.2 Binary logistic regression analysis of primary outcome measures

Using a ≥30% reduction in THI score at the 6-month follow-up as the dependent variable, binary logistic regression showed that age (OR = 0.326, *P* = 0.003) and baseline THI grade (OR = 2.699, *P* = 0.001) were factors associated with meeting response criteria for long-term changes in THI scores. Younger age and more severe baseline tinnitus were associated with greater observed THI score reductions in THI scores (Table 6). The absolute-change sensitivity analysis (≥7 points) confirmed the robustness of the long-term findings: age (OR = 0.375, 95% CI 0.160–0.879, *P* = 0.024) and baseline THI grade (OR = 6.451, 95% CI 2.472–16.837, *P* < 0.001) remained significantly associated with response (Supplementary Table 10).

**Table 6:** Results of long-term changes in THI scores analysis for primary outcome measures.

Variable	Effective group (*n* = 114) *n* (%)	Ineffective group (*n* = 26) *n* (%)	Univariate *P*-value	Multivariate analysis				
				β	SE	OR [Exp (B)]	95% CI for OR	*P*-value
Gender			0.129					
Male	31 (27.2%)	11 (42.3%)						
Female	83 (72.8%)	15 (57.7%)						
Age group			0.044	−1.122	0.379	0.326	0.155–0.684	0.003
≤39 years	46 (40.4%)	4 (15.4%)						
40–59 years	51 (44.7%)	15 (57.7%)						
≥60 years	17 (14.9%)	7 (26.9%)						
THI grade			0.012	0.993	0.310	2.699	1.469–4.958	0.001
Grade II	30 (26.3%)	13 (50.0%)						
Grade III	38 (33.3%)	11 (42.3%)						
Grade IV	28 (24.6%)	1 (3.8%)						
Grade V	18 (15.8%)	1 (3.8%)						
Tinnitus laterality			0.526					
Left ear	29 (25.4%)	9 (34.6%)						
Right ear	32 (28.1%)	5 (19.2%)						
Bilateral	53 (46.5%)	12 (46.2%)						
Disease course			0.232					
≤3 months	45 (39.5%)	7 (26.9%)						
>3 months	69 (60.5%)	19 (73.1%)						
Negative emotions			0.456					
No	44 (38.6%)	8 (30.8%)						
Yes	70 (61.4%)	18 (69.2%)						
Sleep disturbance			0.746					
No	40 (35.1%)	10 (38.5%)						
Yes	74 (64.9%)	16 (61.5%)						
Hearing loss			0.249					
No	43 (37.7%)	13 (50.0%)						
Yes	71 (62.3%)	13 (50.0%)						
Vertigo			0.035	1.388	0.790	4.008	0.852–18.865	0.079
No	83 (72.8%)	24 (92.3%)						
Yes	31 (27.2%)	2 (7.7%)						
Ear fullness and distension			0.427					
No	43 (37.7%)	12 (46.2%)						
Yes	71 (62.3%)	14 (53.8%)						
Hypertension			0.802					
No	76 (66.7%)	18 (69.2%)						
Yes	38 (33.3%)	8 (30.8%)						
Diabetes mellitus			0.210					
No	100 (87.7%)	25 (96.2%)						
Yes	14 (12.3%)	1 (3.8%)						
Rhinitis			0.877					
No	72 (63.2%)	16 (61.5%)						
Yes	42 (36.8%)	10 (38.5%)						

#### 3.4.3 Binary logistic regression analysis of secondary outcome measures

Using a ≥1-grade reduction in THI grade at the 6-month follow-up as the dependent variable, binary logistic regression showed that baseline THI grade (OR = 4.455, *P* < 0.01) and age (OR = 0.266, *P* = 0.001) were factors associated with meeting response criteria for long-term changes in THI scores. For each 1-grade increase in THI grade, the probability of meeting response criteria increased to 4.455 times; for each 1-group increase in age, the probability of meeting response criteria decreased to 0.266 times. Younger patients and those with severe tinnitus had greater observed THI score reductions (Table 7).

**Table 7:** Results of long-term changes in THI scores analysis for secondary outcome measures.

Variable	Effective group (*n* = 116) *n* (%)	Ineffective group (*n* = 24) *n* (%)	Univariate *P*-value	Multivariate analysis				
				β	SE	OR [Exp (B)]	95% CI for OR	*P*-value
Gender			0.378					
Male	33 (28.4%)	9 (37.5%)						
Female	83 (71.6%)	15 (62.5%)						
Age group			0.032	−1.325	0.416	0.266	0.118–0.600	0.001
≤39 years	47 (40.5%)	3 (12.5%)						
40–59 years	51 (44.0%)	15 (62.5%)						
≥60 years	18 (15.5%)	6 (25.0%)						
THI grade			0.000	1.494	0.387	4.455	2.086–9.517	<0.001
Grade II	27 (23.3%)	16 (66.7%)						
Grade III	43 (37.1%)	6 (25.0%)						
Grade IV	28 (24.1%)	1 (4.2%)						
Grade V	18 (15.5%)	1 (4.2%)						
Tinnitus laterality			0.334					
Left ear	29 (25.0%)	9 (37.5%)						
Right ear	33 (28.4%)	4 (16.7%)						
Bilateral	54 (46.6%)	11 (45.8%)						
Disease course			0.176					
≤3 months	46 (39.7%)	6 (25.0%)						
>3 months	70 (60.3%)	18 (75.0%)						
Negative emotions			0.968					
No	43 (37.1%)	9 (37.5%)						
Yes	73 (62.9%)	15 (62.5%)						
Sleep disturbance			0.841					
No	41 (35.3%)	9 (37.5%)						
Yes	75 (64.7%)	15 (62.5%)						
Hearing loss			0.522					
No	45 (38.8%)	11 (45.8%)						
Yes	71 (61.2%)	13 (54.2%)						
Vertigo			0.053					
No	85 (73.3%)	22 (91.7%)						
Yes	31 (26.7%)	2 (8.3%)						
Ear fullness and distension			0.101					
No	42 (36.2%)	13 (54.2%)						
Yes	74 (63.8%)	11 (45.8%)						
Hypertension			0.956					
No	78 (67.2%)	16 (66.7%)						
Yes	38 (32.8%)	8 (33.3%)						
Diabetes mellitus			0.756					
No	104 (89.7%)	21 (87.5%)						
Yes	12 (10.3%)	3 (12.5%)						
Rhinitis			0.614					
No	74 (63.8%)	14 (58.3%)						
Yes	42 (36.2%)	10 (41.7%)						

## 4 Discussion

In this single-center retrospective cohort study, continuous symptom improvement was observed in patients with primary tinnitus during the 6-month follow-up after standardized combined acupuncture, moxibustion, and cupping therapy intervention. Meanwhile, baseline THI grade and age were identified as factors associated with treatment response for long-term changes in THI scores.

### 4.1 Short-term and long-term changes in THI Scores after the combined TCM intervention

The present study showed that the proportion of patients meeting response criteria increased over time after the combined acupuncture, moxibustion, and cupping intervention: judged by a ≥30% reduction in THI score, the response proportion increased from 57.1% to 81.4%; judged by a ≥1-grade reduction in THI grade, the response proportion rose from 63.6% to 82.9%. The observed THI score reductions after the combined TCM intervention persisted and continued to increase over the follow-up period, suggesting a pattern of sustained or delayed symptom improvement that warrants exploration of potential underlying mechanisms.

The mechanisms by which acupuncture-based interventions may influence tinnitus symptoms have been investigated in preclinical and mechanistic studies, though direct evidence in human tinnitus populations remains limited. Below, we summarize several hypothetical pathways that have been proposed in the broader literature as possible explanatory frameworks.

First, the delayed regulatory effect of central neural plasticity. As a physical stimulus, the combined intervention, with acupuncture as its core component, transmits signals to the central nervous system through peripheral nerves potentially influencing abnormal neuronal discharge and plasticity remodeling in the auditory cortex. Animal experiments in adult mouse models suggest that acupuncture may reverse abnormal neural maps by promoting cortical surface plasticity (e.g., synaptic remodeling and neurogenesis), with this process requiring 4–6 weeks in mice (23, 24), the temporal correspondence between 4–6 weeks in mice and 6 months in humans is speculative and not empirically established; it is presented here only as a theoretical parallel. Whether analogous processes occur in humans and whether they translate to clinically meaningful symptom changes remain entirely speculative.

Second, the remodeling of dynamic functional connectivity (dFC) stability. Studies using resting-state fMRI in other tinnitus cohorts have reported that acupuncture may reduce temporal variability of dFC (e.g., reduced fluctuation of connectivity between the auditory cortex and the default mode network), potentially promoting a shift from 'disordered and unstable' to 'ordered and stable' network states. Some authors have hypothesized that this process may require more than 6 months (25). While this temporal hypothesis is consistent with our observation of increased response rates at 6-month follow-up, this temporal association alone does not establish causality. No neuroimaging data were obtained in this cohort, and the observed symptom trajectory may equally reflect natural history, habituation, or regression to the mean.

Third, the modulation of cortical hemodynamic activity and neural excitation-inhibition balance. A preliminary fNIRS study in 18 patients with bilateral subjective tinnitus reported altered oxyhemoglobin concentration in the left auditory cortex following a 10-session combined TCM intervention, though changes in the right auditory cortex and during sound stimulation were not significant (26). The authors speculated that this hemodynamic response might reflect neuroplastic changes or normalization of excitation-inhibition balance, though this interpretation remains hypothetical and the clinical relevance of these small hemodynamic shifts is unclear. No fNIRS, electrophysiological, or biomarker measurements were performed in the present cohort, and we cannot attribute our observed symptom trajectory to these or any other neurobiological mechanisms.

Collectively, these mechanistic investigations provide preliminary leads but do not constitute established evidence for any specific mechanism of action.

### 4.2 Analysis of influencing factors for short-term changes in THI scores

It is important to note that 37.1% of our cohort had tinnitus duration ≤ 3 months, which falls within the acute phase according to the German S3 Guideline (2). We performed a sensitivity analysis by excluding patients with acute tinnitus to assess the robustness of the findings. In the analysis of the full sample, age and baseline THI grade were correlated with short-term symptom improvement. However, after sensitivity adjustment, both factors no longer showed statistically significant associations with short-term outcomes (age: OR = 0.683, 95%CI 0.378–1.236, *P* = 0.208; THI grade: OR = 1.486, 95%CI 0.927–2.380, *P* = 0.100). Sensitivity analysis revealed minimal inter-individual differences in treatment response at the short-term post-treatment time point, with most patients achieving comparable symptom improvement. During long-term follow-up, younger patients and those with higher baseline THI grades exhibited greater observed THI score reductions.

### 4.3 Analysis of influencing factors for long-term changes in THI scores

The association between younger age and greater observed THI score reduction at 6-month follow-up warrants cautious interpretation. In the broader literature, age-related decline in central neural plasticity has been well documented (27). And tinnitus pathogenesis has been hypothesized to involve maladaptive neuroplastic changes and aberrant functional reorganization within auditory and non-auditory networks, based on these observations, some authors have speculated that younger patients—who may retain greater neuroplastic capacity—could show more favorable responses to interventions that are thought to engage plastic processes, including acupuncture-based therapies (28). However, this mechanistic chain remains entirely hypothetical in the context of the present study.

For patients with higher baseline THI scores, the observed association with treatment response can be primarily explained by methodological considerations. First, the mathematical properties of the THI scale create greater room for improvement in patients with more severe baseline handicap (29). The THI ranges from 0 to 100, meaning that a patient with a baseline score of 90 points has a theoretical maximum reduction of 90 points, whereas a patient with a baseline score of 30 points is limited to a maximum 30-point reduction even with complete symptom resolution. This mathematical coupling—where the percentage-change outcome is inherently baseline-dependent—likely contributes to the observed association. Second, the phenomenon of regression to the mean may also play a role: patients with more extreme baseline scores tend to show greater absolute and relative improvement upon repeated measurement, regardless of treatment effect. This is particularly relevant in a condition like tinnitus, which is known for spontaneous fluctuation over time.

While some preclinical and mechanistic studies have suggested associations between tinnitus severity and neurofunctional changes or inflammatory markers in other research contexts (30, 31). We did not measure any of these parameters in this cohort. Therefore, we cannot confirm, refute, or attribute our observed associations to any specific biological process. The association between baseline THI grade and treatment response should be interpreted as a statistical finding that may be influenced by baseline-dependent measurement properties, rather than evidence of differential biological responsiveness to the intervention.

### 4.4 Generalizability of the combined TCM intervention treatment

Gender, tinnitus laterality, disease course, accompanying emotions, sleep disorders and other factors had no association with meeting response criteria, indicating that the combined TCM intervention may be a feasible intervention for a broad range of patients with primary tinnitus.

### 4.5 Observed associations and clinical implications

Our results suggest that younger patients and those with higher baseline THI severity tend to experience greater reductions in tinnitus-related handicap after receiving this standardized combined TCM intervention. Clinically, young patients and those with severe baseline tinnitus showed greater observed THI score reductions in this observational study; however, these findings should be interpreted cautiously given the study design. The observed continued increase in THI score reductions over the 6-month follow-up period warrants further investigation. This study showed that the observed THI score reductions continued to increase after the end of the intervention period, and the 6-month follow-up response proportion was significantly higher than that immediately after treatment. It is suggested that clinical practice should not only evaluate short-term changes in THI scores, but adhere to long-term follow-up of at least 6 months to fully reflect the sustained changes in THI scores observed after the intervention.

The standardized combined TCM intervention protocol using Tinggong, Tinghui, and Yifeng was associated with observed THI score reductions in this single-center cohort. Gender, tinnitus laterality, and accompanying symptoms were not significantly associated with meeting response criteria in this study. However, these observations should not be interpreted as evidence of efficacy, safety, or generalizability, given the single-center uncontrolled retrospective design, the lack of a comparison group, and the potential influence of unrecorded concomitant treatments.

### 4.6 Study limitations and prospects

#### 4.6.1 This study has several limitations

First, this is a single-center retrospective cohort study without a sham, blank or usual-care control group. Therefore, we cannot rule out the influences of the natural course of tinnitus, spontaneous symptom fluctuation, regression to the mean and patient habituation on THI score changes. Second, routine medical records did not fully document patients' concomitant interventions including medications, hearing aids and sound therapy during the study period, which may act as unmeasured confounding factors. Third, the single-center design and inclusion of only treatment completers may lead to selection bias and limit generalizability. Fourth, we did not conduct stratification based on TCM syndrome patterns, and the underlying mechanisms remain to be further explored. In the future, multi-center, large-sample randomized controlled trials with strict control of confounding factors and standardized recording of concomitant treatments are warranted to further explore the effects of the combined TCM intervention on tinnitus. Fifth, the mechanistic discussions in Section 4.1 are presented as background hypotheses from the broader literature, not as mechanisms established or supported by the present data. Since neuroimaging, electrophysiology, inflammatory biomarkers, and microcirculation indicators were not measured in this cohort, we cannot confirm, refute, or attribute any observed symptom changes to these processes. The temporal association between treatment and symptom trajectory does not imply causation or specific biological mechanism. Sixth, this study evaluated a combined TCM intervention protocol comprising acupuncture, moxibustion, and cupping. The retrospective, real-world design and the standardized combined protocol preclude determination of the individual contributions of each component to the observed outcomes. Future randomized controlled trials with factorial designs or component-dismantling arms are warranted to elucidate the specific contributions of the combined TCM intervention, moxibustion, and cupping. In addition, this study only included patients who completed the full combined TCM intervention course and 6-month follow-up. Nineteen patients discontinued treatment before completion, while baseline information was available for these non-completers, post-treatment THI scores were not obtained. We did not perform a sensitivity analysis treating non-completers as non-responders using all 159 treatment initiators as the denominator. Consequently, this analysis represents a completers-only analysis, which may introduce selection bias and affect the generalizability of our findings. What's more, multiple statistical tests were performed for different outcomes and time points, which may increase the risk of type I error. We therefore interpreted all *P* values with great caution.

## 5 Conclusion

In this single-center retrospective cohort study, continued reductions in THI scores were observed over 6 months among patients with primary tinnitus who received a standardized combined acupuncture, moxibustion, and cupping protocol. Baseline THI grade and age were factors associated with meeting response criteria: younger patients and those with more severe baseline tinnitus showed greater observed THI score reductions.

## Data Availability

The original contributions presented in the study are included in the article/Supplementary material, further inquiries can be directed to the corresponding authors.
