# Supplementary material for: 6-month symptom changes and factors associated with treatment response following combined acupuncture, moxibustion, and cupping protocol in patients with primary tinnitus: a retrospective cohort study
Source: Front Neurol. 2026 Jul 14;17:1869226. doi: 10.3389/fneur.2026.1869226 (PMC13408240; doi:10.3389/fneur.2026.1869226)
Supplement: Supplementary file 1 [file Supplementary_file_1.docx]

Supplementary Material

**Table8 Sensitivity Analysis Excluding tinnitus duration ≤3 months(n=88)**

| Outcome | Time point | Variable | Main analysis OR (95% CI) | P | Sensitivity analysis OR (95% CI) | P |
| --- | --- | --- | --- | --- | --- | --- |
| Primary (THI reduction ≥30%) | Short-term | Age | 0.577 (0.353-0.943) | 0.028 | 0.683 (0.378–1.236) | 0.208 |
|  |  | Baseline THI grade | — | — | 1.151 (0.740–1.791) | 0.533 |
|  | Long-term | Age | 0.326 (0.155-0.684) | 0.003 | 0.306 (0.132–0.717) | 0.006 |
|  |  | Baseline THI grade | 2.699 (1.469-4.958) | 0.001 | 2.829 (1.278–6.266) | 0.008 |
| Secondary (THI reduction ≥1-grade) | Short-term | Age | — | — | 0.655 (0.356–1.208) | 0.175 |
|  |  | Baseline THI grade | 1.662 (1.144-2.415) | 0.008 | 1.486 (0.927–2.380) | 0.1 |
|  | Long-term | Age | 0.266 (0.118-0.600) | 0.001 | 0.230 (0.087–0.611) | 0.003 |
|  |  | Baseline THI grade | 4.455 (2.086-9.517) | 0 | 4.706 (1.857–11.929) | 0.001 |

**Table9 Sensitivity Analysis Excluding age ≤18 years(n=138)**

| Outcome | Time point | Variable | Main analysis OR (95% CI) | P | Sensitivity analysis OR (95% CI) | P |
| --- | --- | --- | --- | --- | --- | --- |
| Primary(THI reduction ≥30%) | Short-term | Age | 0.577 (0.353-0.943) | 0.028 | 0.564 (0.341–0.935) | 0.026 |
|  |  | Baseline THI grade | — | — | 1.281 (0.903–1.818) | 0.166 |
|  | Long-term | Age | 0.326 (0.155-0.684) | 0.003 | 0.336 (0.162–0.700) | 0.004 |
|  |  | Baseline THI grade | 2.699 (1.469-4.958) | 0.001 | 2.886 (1.575–5.290) | 0.001 |
| Secondary(THI reduction≥1-grade) | Short-term | Age | — | — | 0.545 (0.297–1.000) | 0.05 |
|  |  | Baseline THI grade | 1.662 (1.144-2.415) | 0.008 | 2.085 (1.340–3.244) | 0.001 |
|  | Long-term | Age | 0.266 (0.118-0.600) | 0.001 | 0.413 (0.180–0.944) | 0.036 |
|  |  | Baseline THI grade | 4.455 (2.086-9.517) | 0 | 3.595 (1.709–7.565) | 0.001 |

**Table 10.** Comparison of binary logistic regression results between relative change (≥30%) and absolute change (≥7 points) criteria at post-treatment and 6-month follow-up.

| Time point | Outcome criterion | Variable | β | SE | P | OR (95% CI) |
| --- | --- | --- | --- | --- | --- | --- |
| Post-treatment | Relative change ≥30%(Primary analysis) | Age | -0.549 | 0.25 | 0.028 | 0.577 (0.353–0.943) |
| (Short-term) | (Primary analysis) | THI grade | — | — | — | — |
|  | Absolute change ≥7 points | Age | -0.363 | 0.276 | 0.188 | 0.696 (0.405–1.194) |
|  |  | THI grade | 0.72 | 0.213 | 0.001 | 2.055 (1.354–3.120) |
| 6-month follow-up | Relative change ≥30%(Primary analysis) | Age | -1.122 | 0.379 | 0.003 | 0.326 (0.155–0.684) |
| (Long-term) |  | THI grade | 0.993 | 0.31 | 0.001 | 2.699 (1.469–4.958) |
|  | Absolute change ≥7 points | Age | -0.98 | 0.434 | 0.024 | 0.375 (0.160–0.879) |
|  |  | THI grade | 1.864 | 0.489 | <0.001 | 6.451 (2.472–16.837) |

**
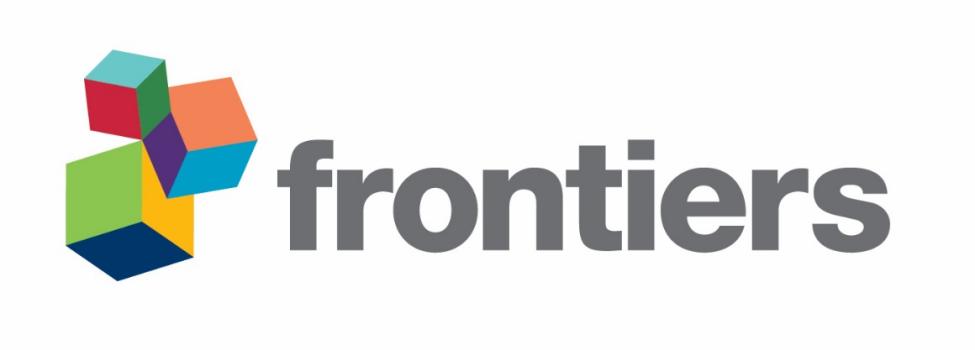
**
