# Supplementary material for: 6-month symptom changes and factors associated with treatment response following combined acupuncture, moxibustion, and cupping protocol in patients with primary tinnitus: a retrospective cohort study
Source: Front Neurol. 2026 Jul 14;17:1869226. doi: 10.3389/fneur.2026.1869226 (PMC13408240; doi:10.3389/fneur.2026.1869226)
Supplement: Supplementary file 2 [file Table_1.docx]

**STROBE Statement—Checklist of items**

| **Section/Topic** | **Item No.** | **Recommendation** | **Reported on page** |
| --- | --- | --- | --- |
| **Title and abstract** | 1a | Indicate the study's design with a commonly used term in the title or the abstract | Title and Abstract |
|  | 1b | Provide in the abstract an informative and balanced summary of what was done and what was found | Abstract |
| **Introduction** | 2 | Explain the scientific background and rationale for the investigation being reported | Introduction |
|  | 3 | State specific objectives, including any prespecified hypotheses | Introduction |
| **Methods** | 4 | Present key elements of study design early in the paper | Methods — Subjects |
|  | 5 | Describe the setting, locations, and relevant dates, including periods of recruitment, exposure, follow-up, and data collection | Methods — Subjects |
|  | 6a | Give the eligibility criteria, and the sources and methods of selection of participants. Describe methods of follow-up | Methods — Inclusion and Exclusion Criteria |
|  | 6b | For matched studies, give matching criteria and number of exposed and unexposed | N/A (unmatched cohort) |
|  | 7 | Clearly define all outcomes, exposures, predictors, potential confounders, and effect modifiers. Give diagnostic criteria, if applicable | Methods — Diagnostic Criteria; Methods — Outcome Measures |
|  | 8 | For each variable of interest, give sources of data and details of methods of assessment (measurement). Describe comparability of assessment methods if there is more than one group | Methods — Diagnostic Criteria; Methods — Outcome Measures |
|  | 9 | Describe any efforts to address potential sources of bias | Methods — Sensitivity Analysis; Discussion — Limitations |
|  | 10 | Explain how the study size was arrived at | Methods — Subjects |
|  | 11 | Explain how quantitative variables were handled in the analyses. If applicable, describe which groupings were chosen and why | Methods — Statistical Analysis |
|  | 12a | Describe all statistical methods, including those used to control for confounding | Methods — Statistical Analysis |
|  | 12b | Describe any methods used to examine subgroups and interactions | Methods — Sensitivity Analysis |
|  | 12c | Explain how missing data were addressed | Methods — Treatment methods; Discussion — Limitations |
|  | 12d | If applicable, describe how loss to follow-up was addressed | Methods — Treatment methods; Methods — Attrition Criteria |
|  | 12e | Describe any sensitivity analyses | Methods — Sensitivity Analysis |
| **Results** | 13a | Report the numbers of individuals at each stage of the study—e.g., numbers potentially eligible, examined for eligibility, confirmed eligible, included in the study, completing follow-up, and analysed | Results — Baseline Characteristics; Figure 1 |
|  | 13b | Give reasons for non-participation at each stage | Methods — Treatment methods; Figure 1 |
|  | 13c | Consider use of a flow diagram | Figure 1 |
|  | 14a | Give characteristics of study participants (e.g., demographic, clinical, social) and information on exposures and potential confounders | Results — Baseline Characteristics; Table 2 |
|  | 14b | Indicate the number of participants with missing data for each variable of interest | Results — Baseline Characteristics |
|  | 14c | Summarise follow-up time—e.g., average and total amount | Methods — Follow-up |
|  | 15 | Report numbers of outcome events or summary measures over time | Results |
|  | 16a | Give unadjusted estimates and, if applicable, confounder-adjusted estimates and their precision (e.g., 95% confidence interval). Make clear which confounders were adjusted for and why they were included | Results; Tables 3–6 |
|  | 16b | Report category boundaries when continuous variables were categorised | Methods — Statistical Analysis |
|  | 16c | If relevant, consider translating estimates of relative risk into absolute risk for a meaningful time period | N/A |
|  | 17 | Report other analyses done—e.g., analyses of subgroups and interactions, and sensitivity analyses | Methods — Sensitivity Analysis |
| **Discussion** | 18 | Summarise key results with reference to study objectives | Discussion |
|  | 19 | Discuss limitations of the study, taking into account sources of potential bias or imprecision. Discuss both direction and magnitude of any potential bias | Discussion — Limitations |
|  | 20 | Give a cautious overall interpretation of results considering objectives, limitations, multiplicity of analyses, results from similar studies, and other relevant evidence | Discussion |
|  | 21 | Discuss the generalisability (external validity) of the study results | Discussion — Generalizability; Discussion — Clinical Implications |
| **Other information(**Funding, p.8) | 22 | Give the source of funding and the role of the funders for the present study and, if applicable, for the original study on which the present article is based | Funding |

**Reference:** *Vandenbroucke JP, von Elm E, Altman DG, Gøtzsche PC, Mulrow CD, Pocock SJ, Poole C, Schlesselman JJ, Egger M; STROBE Initiative. Strengthening the Reporting of Observational Studies in Epidemiology (STROBE): explanation and elaboration. PLoS Med. 2007;4(10):e296.*
