# Supplementary material for: 6-month symptom changes and factors associated with treatment response following combined acupuncture, moxibustion, and cupping protocol in patients with primary tinnitus: a retrospective cohort study
Source: Front Neurol. 2026 Jul 14;17:1869226. doi: 10.3389/fneur.2026.1869226 (PMC13408240; doi:10.3389/fneur.2026.1869226)
Supplement: Supplementary file 3 [file Table_2.docx]

**STRICTA 2010 Checklist**

| Item | Detail | Reported in Manuscript | Details |
| --- | --- | --- | --- |
| **1. Acupuncture rationale** |  |  |  |
| 1a | Style of acupuncture (e.g., Traditional Chinese Medicine, Japanese, Korean, Western medical, Five Element, ear acupuncture, etc.) | Yes | Traditional Chinese Medicine (TCM) style acupuncture |
| 1b | Reasoning for treatment provided, based on historical context, literature sources, and/or consensus methods, with references where appropriate | Yes | Based on TCM theory and clinical practice guidelines; referenced in the manuscript |
| 1c | Extent to which treatment was varied | Yes | Standardized protocol; no variation in acupoint selection or stimulation method across participants |
| **2. Details of needling** |  |  |  |
| 2a | Number of needle insertions per subject per session (mean and range where relevant) | Yes | 3 needle insertions per session for unilateral tinnitus (affected side); 6 needle insertions per session for bilateral tinnitus (both sides) |
| 2b | Names (or location if no standard name) of points used (uni-/bilateral) | Yes | Tinggong (SI19), Tinghui (GB2), and Yifeng (TE17); unilateral for unilateral tinnitus, bilateral for bilateral tinnitus |
| 2c | Depth of insertion, based on a specified unit of measurement, or on a particular tissue level | Yes | 20–30 mm depth |
| 2d | Response sought (e.g., de qi or muscle twitch response) | Yes | De qi sensation (soreness, fullness, or radiation toward the ear) |
| 2e | Needle stimulation (e.g., manual, electrical) | Yes | Moxibustion and retroauricular flash cupping were applied as standardized adjunctive therapies after needling. No electroacupuncture, laser acupuncture, auricular stimulation, or other adjunctive stimulation was used. |
| 2f | Needle retention time | Yes | 30 minutes |
| 2g | Needle type (diameter, length, and manufacturer or material) | Yes | Sterile 0.30 mm × 50 mm needles (stainless steel) |
| **3. Treatment regimen** |  |  |  |
| 3a | Number of treatment sessions | Yes | 10 sessions per course |
| 3b | Frequency and duration of treatment sessions | Yes | Every other day, 3 sessions per week,The full course take about 4 weeks to complete. |
| **4. Other components of treatment** |  |  |  |
| 4a | Details of other interventions administered to the acupuncture group (e.g., moxibustion, cupping, herbs, exercises, lifestyle advice) | Yes | Moxibustion was applied immediately after needling manipulation. After needle removal, flash cupping was performed on the skin. |
| 4b | Setting and context of treatment, including instructions to practitioners, and information and explanations to patients | YES | We clarified the treatment setting and practitioner requirements. All therapies were performed in the hospital outpatient department following a unified standard operating procedure, and routine precautions were told to all patients. |
| **5.Description of participating acupuncturists** | qualification or professional affiliation, years in acupuncture practice, other relevant experience | YES | All acupuncture, moxibustion and flash cupping procedures were performed by one senior chief physician of traditional Chinese medicine. The practitioner possesses complete professional qualifications, and has decades of clinical experience specializing in acupuncture treatment for ear disorders and tinnitus. All operations were implemented strictly in accordance with the unified standard operating procedure. |
| **6. Control or comparator interventions** |  |  |  |
| 6a | Rationale for the control or comparator in the context of the research question, with sources that justify this choice | N/A | Retrospective cohort study; no control/comparator group |
| 6b | Precise description of the control or comparator. If sham acupuncture or any other type of acupuncture-like control is used, provide details as for Items 1 to 3 above | N/A | Retrospective cohort study; no control/comparator group |

**Reference:** MacPherson H, Altman DG, Hammerschlag R, Li Y, Wu T, White A, Moher D, STRICTA Revision Group. Revised STandards for Reporting Interventions in Clinical Trials of Acupuncture (STRICTA): extending the CONSORT statement. Acupunct Med. 2010;28(2):83-93. doi:10.1136/aim.2010.010405
